# Health literacy in patients with Type 2 Diabetes Mellitus: A systematic review

**DOI:** 10.1016/j.clinsp.2025.100774

**Published:** 2025-09-22

**Authors:** Ariel Cesar de Carvalho, Matheus Tonholo Silva, Isadora Lagreca Garrafa Treptow, Carolina de Oliveira Cruz Latorraca, Rafael Leite Pacheco, Victor Alexandre dos Santos Valsecchi, Rachel Riera, Lucas Leite Cunha

**Affiliations:** a Departament of Medicine, Escola Paulista de Medicina (EPM), Universidade Federal de São Paulo (Unifesp), São Paulo, SP, Brazil; b Departament of Surgery, Escola Paulista de Medicina (EPM), Universidade Federal de São Paulo (Unifesp), São Paulo, SP, Brazil; c Hospital Sírio Libanês, São Paulo, SP, Brazil; d Centro Universitário São Camilo, São Paulo, SP, Brazil; e Discipline of Evidence-Based Medicine, Escola Paulista de Medicina (EPM), Universidade Federal de São Paulo (Unifesp), São Paulo, SP, Brazil; f A.C.Camargo Cancer Center, São Paulo, SP, Brazil; g Universidade Anhembi Morumbi, School of Medicine, São José dos Campos, SP, Brazil

**Keywords:** Health literacy, Diabetes mellitus, Type 2, Diabetes Complications, Diabetes knowledge

## Abstract

**Objective:**

To map studies that assessed the Health Literacy (HL) of people with diabetes, provide a global view of this level of literacy, and investigate its relationship between glycemic target and complication rates.

**Materials and methods:**

The authors conducted a systematic and sensitive search across the following electronic databases, including publications from inception to August 2021: Embase, LILACS, Cochrane Library, and MEDLINE. Any type of study that assessed any clinical question related to HL in adults with Type 2 Diabetes Mellitus (T2DM) was selected. In the study, participants had to fill out questionnaires that assessed their level of HL with one of the following tools: TOFHLA, S-TOFHLA, NVS, and REALM.

**Results:**

A total of 57 records were included in this review. The prevalence of inadequate HL ranged between 4.7% and 71.7%. Twenty-six studies evaluated the association between HL and glycemic target, and four investigated the association between HL rates and diabetes complications. Ten of them found an inverse association between HL and glycated hemoglobin, suggesting that inadequate diabetic control was more frequent among patients with lower levels of HL.

**Conclusions:**

This study found that, apparently, there is a direct relationship between the level of HL and disease control as well as an inverse relationship between HL and the risk of developing some complications. These findings could justify the routine assessment of HL among patients with T2DM, as well as the development of strategies to improve their levels.

## Highlights

• Inadequate health literacy is prevalent (4.7 %–71.7 %) in individuals with Type 2 Diabetes Mellitus (T2DM).
• Lower health literacy correlates with poorer glycemic control (10 studies confirmed an inverse association with HbA1c).
• Increased risk of microvascular complications with inadequate health literacy.
• Socioeconomic factors (e.g., HDI) significantly influence health literacy levels and diabetes outcomes.
• Routine health literacy assessment in T2DM may improve disease control and reduce complications (e.g., retinopathy, neuropathy).

## Introduction

Diabetes Mellitus (DM) affects approximately 382 million people worldwide, and this number is expected to reach 471 million by 20,35.^1^ At the beginning of the 21st century, over 5 % of all world deaths were associated with DM, making it the fifth leading cause of death as well as an important cause of morbidity.^2^ People with DM are at increased risk of cardiovascular events, such as acute myocardial infarction, as well as cerebrovascular disease, amputations, blindness, and end-stage renal disease.^3^ These complications, although preventable, are directly dependent on an adequate management of intermediate risk factors (such as high blood pressure, lipid levels and glycemia), which, together with genetic susceptibility, determine the risks of adverse events in people with DM.^3^^,^^4^ Thus, DM is an important example of a chronic illness, with a high level of complexity that requires extensive self-care, education, and personalized clinical management.^5^ Health literacy is one of the forms of evaluating the relationship between self-care and health education.

Health literacy is defined as the level of capacity of individuals to obtain, process, and understand information related to health and services required for making proper decisions in health.^6^ Low health literacy rates (also known as “inadequate” literacy) are very common worldwide.^7^ More than one-third of Americans have low scores of health literacy and difficulties in addressing health issues satisfactorily; this also poses significant economic problems.^7^^,^^8^

Health literacy includes skills such as reading and understanding texts, locating and interpreting information contained in documents, communicating effectively in health-related subjects, as well as skills for dealing with numerical information and tasks, such as interpreting medication dosages, food labels, and glycemic level measurements.^6^ All these tasks are part of the usual routine of people with DM. Since diabetic self-care is largely based on printed materials and verbal instructions, these specific patients would undoubtedly need to have adequate health literacy levels.^9^

It is important to assess the health literacy levels of people with diabetes because those with more knowledge about their disease are able to better understand and control their condition. Therefore, mapping studies that assessed the health literacy of people with diabetes can provide a global view of this level of literacy and investigate its relationship with glycemic targets and rates of complications.

Thus, this systematic review aimed to map studies assessing health literacy in people with diabetes, estimate the prevalence of inadequate literacy, and examine its relationship with glycemic targets and complication rates.

## Materials and methods

### Study design

This systematic review of literature followed the PRISMA (Preferred Reporting Items for Systematic Review and Meta-analyses),^10^ and the recommendations for first-person language.^11^ The present systematic review was conducted at the Endocrinology Unit of Universidade Federal de São Paulo (Unifesp), São Paulo (SP), Brazil.

### Criteria for including studies

The authors included any type of studies that provided information about health literacy in patients with Type 2 DM (T2DM): descriptive studies, cross-sectional studies (prevalence), observational longitudinal studies (incidence, ecological, case series), observational comparative studies (cohort and case-control), intervention longitudinal studies (clinical trials, single-arm experimental cohorts, before-and-after) and secondary studies (systematic reviews, meta-analyses, economic evaluations).

The authors considered studies addressing any clinical question related to health literacy in adults with T2DM. In the study, participants had to fill out questionnaires that assessed their level of health literacy with one of the following tools for assessing health literacy: TOFHLA (Test of Functional Health Literacy in Adults),^12^ s-TOFHLA (shortened version of TOFHLA),^13^ NVS (Newest Vital Sign)^14^ and REALM (Rapid Estimate of Adult Literacy in Medicine).^15^

Studies that included only participants with type 1 DM were excluded. The authors The authors also excluded studies in which the level of health literacy was a criterion for including or excluding participants.

The authors included studies that analyzed the levels of health literacy in this specific population and its relationship to diabetes control and diabetes complications.

The authors included all the outcomes evaluated by the authors of the included studies, including clinical, laboratory, and administrative/economic outcomes.

### Study outcomes

1) The prevalence of Inadequate Health Literacy worldwide.
2) The relationship between Inadequate Health Literacy and poor glycemic control and diabetes complications.

### Search methods

The authors carried out a wide and sensitive search in the following electronic databases, published from inception until August 28, 2021: Embase (via Elsevier), Latino-American and Caribbean Literature in Health Science ‒ LILACS (via Biblioteca Virtual em Saúde ‒ BVS), *Cochrane Library* (via Wiley), and MEDLINE (via Pubmed). The search strategy is presented in the Supplement Material. There were no language or date of publication restrictions.

### Selection of studies and data extraction methods

The records retrieved from each electronic database were merged into a single list, and duplicates were excluded. The final list of unique records was screened by two independent authors (ACC and ILT or MT) who assessed the eligibility of titles and abstracts. Potentially relevant records were selected for full-text reading and final judgement inclusion. One independent investigator (RR or LL) was invited to resolve disagreements. The selection process was conducted using the Rayyan platform.^16^

Two independent authors also extracted the following data from included studies: Country, study design, study aim, sample size, health literacy tool and scores, association between health literacy and glycemic control, diabetes complications, and other outcomes. Disagreements were also solved by an independent investigator.

### Data extraction and synthesis

The authors used a standardized form created for this review to extract and synthesize the following data from each included study: period and setting, methodological characteristics, participants, and results of the assessed outcomes. Data extraction and synthesis were performed in duplicate by two independent authors (ACC and ILT or MT), and disagreements were resolved by a third author (RR or LL).

### Quality assessment

Study quality was assessed by tools according to study design: the Joanna Briggs Institute checklist for analytical cross-sectional studies (from one to eight points),^17^ the Joanna Briggs Institute checklist for systematic reviews and research syntheses (from one to eleven points),^18^ the Joanna Briggs Institute checklist for qualitative research (from one to ten points),^19^ the Joanna Briggs Institute checklist for quasi-experimental studies (non-randomized experimental studies) (from one to nine points),^20^ and the Cochrane Risk of Bias (RoB) tool for clinical trials.^21^ The results from the Joanna Briggs Institute checklists were summarized by average.

The Joanna Briggs Institute checklists were employed due to their widespread use and comprehensive coverage of various study designs. This choice was made because the checklists offer a practical and flexible framework for assessing methodological quality across diverse research approaches, ensuring comparability without compromising the specific rigor required by each design. Given the diversity of study types utilized in the present research, these checklists were instrumental in standardizing the assessment of methodological quality across studies. They are particularly suitable for this diversity as they provide tailored criteria for different research designs, ensuring that each study is evaluated according to its unique methodological framework. This approach enhances consistency and reduces bias in quality appraisal, making it especially beneficial for integrative reviews involving heterogeneous study types. By using this approach, the authors aimed to ensure a consistent and transparent quality appraisal process, while accommodating the methodological nuances of each study type.

Two authors (ACC and ILT or MT) performed these evaluations. Disagreements were resolved by an independent author (RR or LL).

## Results

### Search outcome

**Fig. 1:**
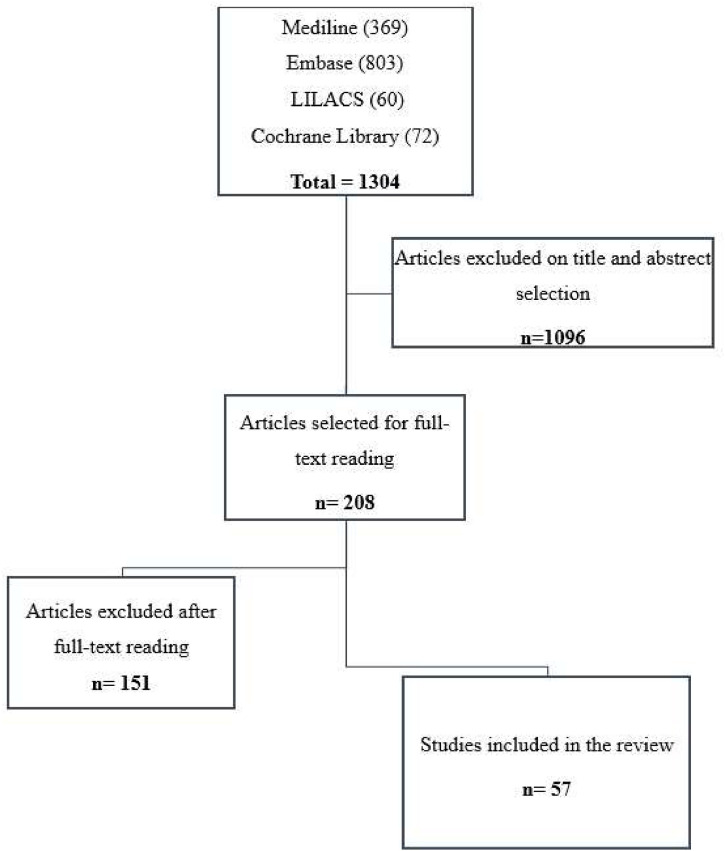
Study identification and selection.

Fig. 1 presents the process of study identification and selection. The initial search retrieved 1304 records. After excluding duplicates and first screening of titles and abstracts, 1096 records were excluded, and 208 were selected for full-text reading. After full-text reading, 151 records were excluded.

LILACS, Latin-American and Caribbean Literature in Health Science.

A total of 57 records fulfilled the selection criteria and were included in this review (three congress abstracts and 54 full-text articles). The authors included 44 cross-sectional studies, two validation studies, two experimental prospective before-and-after studies, one qualitative research, 6 clinical trials, and two systematic reviews.

### Characteristics and main findings of studies included

The total number of participants included was 31,590 and in the 57 included studies was between 26 and 2510 for primary studies, and 2230 and 13,457 in the two systematic reviews.^22^^,^^23^ Most of the studies (29/57, 50.8 %) used the s-TOFHLA tool to assess health literacy. The prevalence of inadequate health literacy ranged between 4.7 % and 71.7 % (Table 1).

**Table 1:** Characteristics, main findings, and quality of studies on health literacy of type 2 DM patients.

Study	Country	HDI	Design	Aim	Sample size	Health literacy tool and scores	Quality
Abduullah et al., 201,9^23^	Malaysia	0.819	Systematic review	To summarize and report evidence on the prevalence of limited HL in people with T2DM globally and on the factors that are associated with the heterogeneity in this prevalence.	29 studies (13,457)	• TOHFLA; s-TOHFLA and similars (23 studies); REALM and similars (5 studies); NVS (1 study); • Global prevalence of limited HL was 34.3 % (95 % CI: 25.8 to 42.8); Prevalence of limited HL in the USA was 28.9 % (95 % CI: 20.4 to 37.3)	11/11 (100 %)
Aljohani et al.; 201,8^47^	Saudi Arabia	0.854	Cross-sectional study	To assess the association between HL level and glycemic control in people with T2DM.	249	s-TOFHLA (0‒100): Mean (SD): 72.0 (16.4) • Adequate: 68.7 % • Moderate: 18 % • Inadequate: 13.3 %	4/8 (50 %)
Almigbal et al., 201,9^46^	Saudi Arabia	0.854	Cross-sectional study	To determine the association and differences between level of HL, presence of depressed mood or anhedonia, and their relationships with diabetes self-management among T2DM people in Saudi Arabia.	352	s-TOFHLA (0‒36): • Adequate (23‒36): 34.1 % • Moderate (18‒22): 16.2 % • Inadequate (0‒17): 49.7 %	6/8 (75 %)
Alvarez et al., 201,8^45^	USA	0.926	Cross-sectional study	To explore the association between health literacy and patient-reported outcomes such as self-monitoring of blood glucose (SMBG), testing, and the clinical outcome of glycemic control as measured by A1C in people with noninsulin-treated T2DM.	448	NVS (0‒6): • Inadequate (< 4):38.17 % • Adequate (≥ 4): 61.83 %	6/8 (75 %)
Ayre et al., 202,1^66^	Australia	0.944	Qualitative research	To address the gap in the literature about health literacy and diabetes self-management by exploring how people with diabetes with varying health literacy levels conceptualize their experiences and efforts to engage in and maintain diabetes self-care behaviours.	26	NVS (0‒6): • Inadequate (< 4):53 % • Adequate (≥ 4): 38 %	10/10 (100 %)
Bains et al., 201,1^24^	USA	0.926	Cross-sectional study	To assess the relationship among HL, knowledge about diabetes, self-care and glycemic control in low-income in patient with diabetes.	125	REALM-R (0‒8) mean (SD): 6.1 (0.3)	6/8 (75 %)
Beverly et al., 201,3^49^	USA	0.926	Randomized controlled trial	To assess the effects of diabetes education on self-care and glycemic control.	134	s-TOFHLA (0‒36): 33.1 (SD = 4.3)	High risk of Bias^a^
Bohanny et al., 201,3^67^	Taiwan	0.912	Cross-sectional study	To assess the relationship among HL, self-efficacy, and self-care behaviors.	150	s-TOFHLA (0‒100): Mean (SD): 76.78 (14.3) • Adequate (67‒100): 76 % • Marginal (54‒66): 17 % • Inadequate (0‒53): 7 %	8/8 (100 %)
Castro et al., 201,4^50^	Brazil	0.765	Cross-sectional study	To assess the level of HL among patients with diabetes managed in a university clinic.	164	s-TOFHLA (0‒36): • Adequate: 73.3 % • Moderate: 2.7 % • Inadequate: 15.3 % (no definition for each level was provided)	6/8 (75 %)
Chen et al., 201,9^52^	Australia	0.944	Cross-sectional study	To describe the level of functional and multidimensional HL among people with diabetes, and evaluate the associations between health literacy and risk factors for diabetic foot disease.	222	s-TOFHLA (0‒36): • Adequate (23‒36): 91.9 % • Moderate (18‒22): 2.7 % • Inadequate (0‒17): 5.4 %	8/8 (100 %)
Chin et al., 202,1^68^	USA	0.926	Cross-sectional study	To examine how HL and its components (processing capacity and knowledge about illness) influence memory for medication purposes.	674	REALM (0‒66): • Adequate (61‒66): 77.74 % • Inadequate (0‒60):22.26 % - 3rd grade and below (0‒18): 1.04 % - 3rd to 6th grade (19‒44): 4.75 % - 7th to 8th grade (45‒60): 16.47 %	8/8 (100 %)
Garcia et al., 201,9^58^	USA	0.926	Cross-sectional study	To identify modifiable psychosocial conditions and social/economic-related factors of low adherence, and to examine sex differences among Mexican heritage adults with T2DM using proportion of days covered as a measure of adherence.	279	NVS (One item question): • Adequate (Correct): 9.0 % • Limited (Incorrect): 91.0 %	4/8 (50 %)
Gordon Singh et al., 201,7^44^	Jamaica	0.734	Cross-sectional study	To determine the relationship between HL levels and health outcomes for people in a type V health center in Western Jamaica.	88	NVS*: • Adequate: 13.6 % • Possibility of Limited HL: 59.1 % • Suggested high likelihood of limited HL: 27.3 % * NVS score was not described.	6/8 (75 %)
Hashim et al., 202,0^43^	Iraq	0.686	Cross-sectional study	To determine the association of HL and nutritional status assessments with glycemic control in adults with T2DM.	280	s-TOFHLA (0‒100): Mean (SD): 45.7 (24.6) • Adequate (67‒100): 23.5 % • Marginal (54‒66): 20.8 % • Inadequate (0‒53): 55.6 %	8/8 (100 %)
Hashim et al., 202,1^69^	Iraq	0.686	Randomized controlled trial	To evaluate the effectiveness of the Simplified Diabetes Nutrition Education (SDNE) on glycemic control and other diabetes-related outcomes in people with T2DM	208	s-TOFHLA (0‒100): Mean (SD): 47.1 (22.7) for nutritional education group and 50.9 (17.6) for control group.	High risk of Bias^a^
Huang et al., 201,8^42^	USA	0.926	Cross-sectional study	To examine the association of HL and medication self-efficacy with self-reported diabetes medication adherence, and the association of health literacy, medication self-efficacy, self-reported diabetes medication adherence with HbA1c among people with T2DM.	174	NVS (0‒6): Mean (SD): 3.7 (2.0) • Inadequate (0‒1): 17.8 % • Marginal (2‒3): 22.4 % • Adequate (≥4): 59.8 %	8/8 (100 %)
Huang et al., 202,0^70^	USA	0.926	Cross-sectional study	To identify which specific factors are associated with diabetes medication adherence differences across various HL levels.	205	NVS (0‒6): Mean (SD): 4.26 (1.73) • Inadequate (< 4): 27.3 % • Adequate (≥ 4): 72.7 %	6/8 (75 %)
Hur et al., 202,0^71^	USA	0.926	Cross-sectional study	To identify potentially modifiable barriers and risks to poor diabetes medication adherence.	756	NVS: not assessed.	4/8 (50 %)
Hussein et al., 201,8^72^	Kuwait	0.803	Cross-sectional study	To estimate the prevalence of health literacy among people with T2DM and investigate its association with several covariates.	359	s-TOFHLA (0‒100): Mean (SD): 56.0 (37.0) • Adequate (67‒100): 35.5 % • Marginal (54‒66): 19 % • Inadequate (0‒53): 45.5 %	6/8 (75 %)
Hussein et al., 202,1^41^	Kuwait	0.803	Cross-sectional study	To investigate the association between health literacy and glycated hemoglobin (HbA1c) among people with T2DM.	356	s-TOFHLA (0‒100): Mean (SD): 56.0 (36.0) • Adequate (67‒100): 35.53 % • Marginal (54‒66): 19.19 % • Inadequate (0‒53): 45.27 %	8/8 (100 %)
Ji M et al., 202,0^40^	China	0.768	Cross-sectional study	To examine the effects of selected personal, behavioral, and environmental factors on self-care behaviors, glycemic control, and metabolic syndrome and T2DM.	207	NVS (0‒6): Mean (SD): 2.0 (2.1)	6/8 (75 %)
Junkhaw et al., 201,9^39^	Thailand	0.800	Cross-sectional study	To evaluate HL levels and determine associated factors among people with diabetes in suburban Bangkok, Thailand.	312	TOHFLA + FCCHL*: Functional HL (0‒30): • Low (0‒14.9): 5.4 % • Moderate (15‒23.9): 64.7 % • High (24‒30): 29.8 % Interactive HL (0‒44): • Low (0‒21.9): 21.5 % • Moderate (22‒35.1): 76.3 % • High (35.2‒44): 2.2 % Critical HL (0‒40): • Low (0‒19.9): 21.2 % • Moderate (20‒31.9): 70.8 % • High (32‒40): 8 % * Adapted, translated, and utilized for this purpose in Thai settings by the Ministry of Public Health.	4/8 (50 %)
Kim et al., 202,0^38^	USA	0.926	Randomized controlled trial	To empirically examine underlying mechanisms of HL role in diabetes management among a group of Korean Americans with T2DM.	250	• REALM (0‒66): Mean (SD): 32.1 (1.5) • DM-REALM (0‒82): Mean (SD): 51.3 (1.7) TOFHLA numeracy subscale (0‒7): Mean (SD): 4.2 (0.2) NVS (0‒6): Mean (SD):1.7 (0.1) The four HL measures were highly correlated with each other, and all correlations were statistically significant. The correlation between REALM and DM-REALM was highest (*r* = 0.91, *p* < 0.001), followed by that for the TOFHLA (*r* = 0.68, *p* < 0.001) and NVS (*r* = 0.47, *p* < 0.001)	High risk of Bias^a^
Kim et al., 201,9^37^	South Korea	0.925	Randomized controlled trial	To evaluate the effects of a social media-based, HL-sensitive diabetes management intervention on patient activation, self-care behaviors, and glucose control compared to telephone-based, HL-sensitive diabetes management intervention and usual care. And to identify how patient HL influenced the effectiveness of HL-sensitive diabetes management interventions.	155	KFHL (Korean REALM adaptation) Low HL (< 6): • Social Media-HL intervention group: 30.8 % • Telephone-HL intervention group: 59.9 % • Control group: 51.9 %	High risk of Bias^a^
Klinovszky et al., 202,1^36^	Hungary	0.854	Cross-sectional study	To explore functional HL levels and numeracy skills in an insulin treated T2DM population are related to diabetes, and their impact on diabetes therapy.	102	s-TOFHLA (0‒36): Mean (SD): 23.78 (10.084) • Adequate (23‒36): 65.3 % • Marginal (17‒22): 6.9 % • Inadequate (0‒16): 27.7 %	6/8 (75 %)
Mann et al., 201,9^48^	USA	0.926	Cross-sectional study	To evaluate the role of HL in attaining adequate glycemic control of T2DM and whether this has any impact on long-term complications of diabetes.	170	NVS (0‒6): • Inadequate (0‒2): 71.76 % • Adequate (3‒6): 28.24 %	6/8 (75 %)
Mashi et al., 201,9^35^	Saudi Arabia	0.854	Cross-sectional study	To identify the prevalence of HL among adult Saudi T2DM patients and determine the clinical factors that are associated with health literacy scores including glycemic control.	249	s-TOFHLA (0‒100): • Adequate (67‒100): 69.9 % • Marginal (54‒66): 16.9 % • Inadequate (0‒53): 13.3 %	8/8 (100 %)
Mayberry et al., 201,4^73^	USA	0.926	Cross-sectional study	To assess the relationship between obstructive family behaviors and glycemic control in patients with high or low health literacy levels.	314	s-TOFHLA (0‒36) mean (SD): 26 (10.8) • Limited (0‒23): 26 % • Suitable (24‒36): 74 %	7/8 (87.5 %)
Mehzabin et al., 201,9^34^	Bangladesh	0.661	Cross-sectional study	To assess the relationship between functional HL and glycemic control of 'patient with diabetes’ among the urban population.	200	s-TOFHLA (0‒36): • Adequate (23‒36): 24.0 % • Marginal (17‒22): 15.5 % • Inadequate (0‒16): 60.5 %	8/8 (100 %)
Nurss et al., 199,7^57^	USA	0.926	Cross-sectional study	To assess the HL in an African American population with diabetes.	131	TOFHLA (0‒100) mean (SD): 57.3 (26.1)	8/8 (100 %)
Osborn et al., 20,10^9^	USA	0.926	Cross-sectional study	To assess the relationship between HL, determinants of self-care in diabetes and glycemic control.	130	REALM: • Limited (0‒6): 29.7 % • Suitable (7‒8): 70.3 %	8/8 (100 %)
Pashaki et al., 201,9^22^	Iran	0.797	Systematic review	To estimate the prevalence of HL among Iranian patients with T2DM.	8 studies (2230)	TOFHLA: • Adequate: 29.72 % (95 % CI: 22.79‒36.64) • Moderate: 26.34 % (95 % CI: 19.49‒33.19) • Inadequate: 43.47 % (95 % CI: 31‒55.95)	11/11 (100 %)
Presley et al., 201,9^74^	USA	0.926	Cross-sectional study	To evaluate factors associated with antidepressant use in a low-income, primarily uninsured, racially and ethnically diverse sample of patients with T2DM to identify patients at risk for untreated depression.	403	s-TOFHLA (0‒36): • Adequate (23‒36): 84 % • Inadequate or Marginal (0‒22): 16 %	8/8 (100 %)
Quartuccio et al., 201,8^33^	USA	0.926	Cross-sectional study	To evaluate the relationship between HL and diabetes and hyperglycemia in older adults, and if this relationship differs between sexes.	2510	REALM (0‒66): • Adequate (61‒66): Men with T2DM:65.5 %; Men with no T2DM: 73.7 %. Women with T2DM: 64.4 %; Women with no T2DM: 83.1 % • Moderate (45‒60): Men with T2DM: 24.4 %; Men with no T2DM: 17.0 %. Women with T2DM: 20.9 %; Women with no T2DM: 10.8 % • Inadequate (0‒44): Men with T2DM: 10.1 %; Men with no T2DM: 9.3 %. Women with T2DM: 14.7 %; Women with no T2DM: 6.1 %	8/8 (100 %)
Rua Castro et al., 202,0^75^	Portugal	0.864	Experimental prospective before-and-after	To know the effect of a health education program on the knowledge of diabetes, self-care training and HL in elderly people with T2DM.	40	NVS (0‒6): • Adequate (4‒6): Men before intervention: 15.8 %; Men after intervention: 26.3 %. Women before intervention: 0 %; Women after intervention: 14.3 %. • Moderate (2‒3): Men before intervention: 21.1 %; Men after intervention: 57.9 %. Women before intervetion:19 %; Women after intervention: 66.7 % • Inadequate (0‒1): Men before intervention: 63.1 %; Men after intervention: 15.8 %. Women before intervention: 81 %; Women after intervention: 19 %	6/9 (66.6 %)
Saeed et al., 201,8^32^	Pakistan	0.557	Cross-sectional study	To evaluate the functional health literacy of patients with T2DM in Lahore and its impact on glycemic control.	204	s-TOFHLA (0‒36): • Adequate (23‒36): 15.2 % • Marginal (17‒22): 17.6 % • Inadequate (0‒16): 67.2 %	8/8 (100 %)
Santos et al., 201,6^51^	Brazil	0.765	Cross-sectional study	To evaluate the conditions of HL of an elderly group.	114	s-TOFHLA (0‒100): • Adequate (67‒100): 15.8 % • Marginal (54‒66): 10.5 % • Inadequate (0‒53): 73.7 %	5/8 (62.5 %)
Sarkar et al., 200,6^76^	USA	0.926	Cross-sectional study	To assess the relationship between self-efficacy and self-care behaviors in patient with diabetes with different levels of HL.	408	s-TOFHLA (0‒36): • Adequate (23‒36): 48.5 % • Moderate (17‒22): 13.25 % • Inadequate (0‒16): 38.25 %	8/8 (100 %)
Sarkar et al., 201,1^77^	USA	0.926	Validation study	To examine the performance of three self-reported HL questions individually and as a summative scale among English and Spanish-speaking, diverse, low-income, populations with T2DM.	296	s-TOFHLA (0‒36): • Adequate (23‒36): 41.2 % • Moderate (17‒22): 11.5 % • Inadequate (0‒16): 47.3 %	8/8 (100 %)
Sayah et al., 201,4^78^	USA	0.926	Cross-sectional study	To examine the measurement properties of the 16 Screening Questions (16-SQ) of inadequate Health Literacy (HL) and their briefer version (3-SQ), and identify the best screen for inadequate HL in non-white populations.	378	s-TOFHLA (0‒36): • Adequate (23‒36): 90.25 % • Moderate (17‒22): 5.03 % • Inadequate (0‒16): 4.72 %	8/8 (100 %)
Schillinger et al., 20,02^6^	USA	0.926	Cross-sectional study	To assess the relationship between health literacy and diabetes outcomes.	408	s-TOFHLA (0‒36) mean: 21	8/8 (100 %)
Schillinger et al., 200,4^79^	USA	0.926	Cross-sectional study	To assess the relationship between health literacy and the quality in doctor patient with diabetes communication.	408	s-TOFHLA (0‒36) mean: 21	8/8 (100 %)
Shigaki et al., 201,0^25^	USA	0.926	Cross-sectional study	To examine the relationship between autonomous motivation and diabetes self-care activities among individuals with diabetes.	77	• REALM (15‒66) mean (SD): 62.2 (8.8) • NVS (max 6) mean (SD): 3.7 (2.0)	8/8 (100 %)
Shiyanbola et al., 201,8^59^	USA	0.926	Cross-sectional study	To examine the association between HL, beliefs in medicines, illness perceptions, and medication adherence in individuals with T2DM and the moderating effects of health literacy (including numeracy and document literacy) on the relationship between illness perceptions, beliefs in medicines, and medication adherence.	174	NVS (0‒6): • Adequate (≥4): 59.8 % • Marginal (2‒3): 22.4 % • Inadequate (0‒1): 17.8 %	6/8 (75 %)
Souza et al., 202,0^31^	Brazil	0.765	Cross-sectional study	To investigate the association between inadequate functional HL and glycemic control in elderly patients with T2DM, and to examine this association in low social support settings, according to Medical Outcomes Study.	166	SAHLPA (0‒18) (Brazilian REALM adaptation): Mean (SD): 13.3 (5.0) • Adequate (14‒18): 53.6 % • Inadequate (0‒13): 46.4 %	6/8 (75 %)
Swavely et al., 201,4^55^	USA	0.926	Experimental prospective before-and-after	To assess the effects of a diabetes educational program on diabetes knowledge, self-efficacy, self-care and metabolic control.	106	S-TOFHLA (0‒36): • Adequate (23‒36): 63.2 % • Moderate (17‒22): 11.3 % • Inadequate (0‒16): 25.5 %	6/9 (66.6 %)
Tan et al., 202,0^30^	Malaysia	0.819	Cross-sectional study	To examine the HL levels among adults with T2DM in the public primary care health clinics within the Klang Health District, factors associated with limited HL and analyze and evaluate if an association between HL and glycemic control can be found.	289	NVS (0‒6): • Adequate (≥4): 17.0 % • Marginal (2‒3): 18.7 % • Inadequate (0‒1): 64.3 %	6/8 (75 %)
Tang et al., 200,8^26^	China	0.768	Cross-sectional study	To assess the relationship between health literacy, diabetes awareness, and diabetes control. To validate the Chinese version of s-TOFHLA.	149	s-TOFHLA: Not assessed	8/8 (100 %)
Taylor et al., 201,3^29^ (abstract)	Australia	0.944	Cross-sectional study	To assess the relationship between HL and glycemic control.	125	TOFHLA (0‒100): mean 79.6	NA
Thurston et al., 201,5^28^	USA	0.926	Cross-sectional study	To assess the relationship between HL and compliance to medication.	192	s-TOFHLA (0‒36) mean (SD): 25.5 (10.5) • Suitable (23‒36): 67 % • Limited (0‒22): 32.8 %	6/8 (75 %)
Tseng et al., 201,7^27^	Taiwan	0.912	Cross-sectional study	To assess the role of knowledge and stages of change (SOC) as serial mediators linking HL to glycemic control.	232	NVS (0‒6): • Adequate (4‒6): 23.7 % • Limited possible (2‒3): 28.9 % • Limited (0‒1): 47.4 %	8/8 (100 %)
Tseng et al., 201,8^80^	Taiwan	0.912	Validation study	To develop a traditional Chinese version of the NVS (NVS-TC) and assess its feasibility, reliability, and validity in Taiwanse patients with T2DM	232	NVS (0‒6): • Adequate (≥4): 23.7 % • Marginal (2‒3): 28.9 % • Inadequate (0‒1): 47.4 %	8/8 (100 %)
Walker et al., 201,4^56^	USA	0.926	Cross-sectional study	To assess the relationship between socioeconomic and psychosocial determinants and diabetes knowledge, self-care, diabetes outcomes and quality of life.	615	s-TOFHLA: Not assessed	8/8 (100 %)
Weinger et al., 201,2^81^ (abstract)	USA	0.926	Randomized controlled trial	To assess the effects of diabetes education on glycemic control.	134	TOFHLA: Assessed but not reported.	NA
White et al., 201,6^82^	USA	0.926	Cross-sectional study	To examine the association of medical mistrust with perceptions of communication quality with providers for diabetes patients enrolled in a clinical trial.	410	S-TOFHLA (0‒36): • Adequate (23‒36): 83 % • Inadequate + marginal (0‒22): 17 %	8/8 (100 %)
White et al., 202,0^83^	USA	0.926	Randomized controlled trial	To compare two distinct care approaches: one based on content from the National Diabetes Education Program (NDEP) and one centered on addressing issues of health communication (PRIDE) to evaluate the potential benefits of focusing on health communication issues in this vulnerable population.	364	S-TOFHLA (0‒36): Mean: 34 (12‒36).	High risk of Bias^a^

HDI, Human Development Index^85^; HL, Health literacy; KFHL, Korean Functional Health Literacy; NVS, Newest Vital Sign; REALM, Rapid Estimate of Adult Literacy in Medici;n^4^ REALM-R, Revised REALM; SAHLPA, Short Assessment of Health Literacy for Portuguese-speaking Adults; SD, Standard Deviation; s-TOFHLA, shortened version of TOFHLA; T2DM, Type 2 Diabetes Mellitus; TOFHLA, Test of Functional Health Literacy in Adults.

a The domains bias in selection of participants into the study, bias in classification, and bias in selection of the reported judged as high risk of bias by the Cochrane RoB tool.

Twenty-six studies evaluated the association between health literacy and glycemic target^6^^,^^9^^,^^24^, ^25^, ^26^, ^27^, ^28^, ^29^, ^30^, ^31^, ^32^, ^33^, ^34^, ^35^, ^36^, ^37^, ^38^, ^39^, ^40^, ^41^, ^42^, ^43^, ^44^, ^45^, ^46^, ^47^ and three investigated the association between health literacy rates and diabetes complications.^6^^,^^32^^,^^48^ Thirty-one addressed the relationship between health literacy and other outcomes, such as difficulty in reading medical texts, interpersonal relationships, self-efficacy, physical activity, diet follow-up, social support, income, age, knowledge about diabetes, and adherence to subjective and objective medication (Table 2).

**Table 2:** Relation between health literacy scores and outcomes in 49 studies involving patients with type 2 DM.

Study	Health literacy and glycemic control	Health literacy and diabetes complications/other outcomes
Aljohani et al.; 201,8^47^	Glycemic control: there was no direct correlation between health literacy and glycemic control with Hb1ac (SD) 8.2 (2.1), 8.3(2.0), 8.3 (2.2) for Inadequate HL, Moderate HL and Adequate HL respectively (*p* > 0.05).	NA
Almigbal et al., 201,9^46^	Glycated hemoglobin: there is no significant relation between low functional HL and level of HbA1c (OR = 0.97, 95 % CI 0.55 to 1.69, *p* = 0.920).	NA
Alvarez et al., 201,8^45^	Participants with limited health literacy had significantly poorer glycemic control than participants with adequate HL: Hb1aC (SD) 7.7 (1.1) vs. 7.5 (1.0), *p* = 0.016).	• There was no statistically significant difference in the frequency of which physicians told their patients to conduct SMBG testing between limited and adequate health literacy groups (*p* = 0.68). • Significantly more patients with limited health literacy performed once-daily SMBG testing (once-daily SMBG testing 49.3 vs. 30.7 %, *p* = 0.001) compared to individuals with adequate health literacy.
Ayre et al., 202,1^66^	NA	Was not observed any notable HL patterns across two monitoring schemas studied, 'Monitoring to increase self-management awareness' and “Monitoring to sustain motivation”. However, the schema “Monitoring to sustain motivation”, was more apparent in the accounts of participants who had adequate HL.
Bains et al., 201,1^24^	Glycemic target: no correlation (95 % CI −0.19 to 0.13).	• Knowledge about diabetes: positive relation through bivariate (*p* < 0.0001) and multivariate analysis (95 % CI 0.29 to 0.82), • Medication taking: no correlation • Self-care of diabetes (diet, exercise, capillary glucose measurement, foot care): no correlation
Bohanny et al., 201,3^67^	NA	• Employee subjects: Higher levels of health literacy were found in patients who were employed at the time of the study (*p* = 0.008). • Age: inverse proportion rate related to health literacy (*p* = 0.004). • Self-effectiveness: positive relation through bivariate analysis (*p* < 0.003)
Castro et al., 201,4^50^	NA	• Afro-Brazilians: a quarter of Afro-Brazilians had inadequate levels of health literacy. • Occupational and educational level: positive correlation. • Age, ethnic group, education and occupation predicted s-TOFHLA (*p* < 0.0001).
Chen et al., 201,9^52^	NA	• Each unit increase in S-TOFHLA scores was associated with 4 % lower odds of being in a higher risk category for foot disease (OR = 0.96, 95 % CI 0.93 to 0.99). However, this association did not persist after adjusting for age, sex and other covariates. • There were no associations between any measures of health literacy and peripheral arterial disease or foot deformity.
Chin et al., 202,1^68^	NA	• Age, processing capacity, knowledge about diabetes and HL were all associated with memory for medication purpose (*p* < 0.001). • Age, education, processing capacity, and knowledge about diabetes were all significantly associated with HL (*p* < 0.001).
Garcia et al., 201,9^58^	NA	There were no associations between health literacy level and medication adherence measures.
Gordon Singh et al., 201,7^44^	Pearson's correlation indicated that health literacy level was not related to the control of random blood glucose (*p* = 0.771)	Pearson's correlation indicated that health literacy level was not related to acute complications (*p* = 0.376) and chronic complications (*p* = 0.620).
Hashim et al., 202,0^43^	Respondents with inadequate HL had a significantly higher HbA1c levels (Mean (SD) = 10.6 (2.6 %)) than those with marginal and adequate HL (*p* < 0.0001).	Respondents with adequate HL had significantly better total cholesterol levels (mean (SD) = 9.4 (2.6 mmoL/L)), LDL - cholesterol (mean (SD) = 6.4 (2.1 mmoL/L), and systolic blood pressure (mean (SD) = 135.3 (25.8 mmHg), than those with marginal and inadequate HL.
Huang et al., 201,8^42^	HL did not have a significant association with HbA1c in bivariate correlation (*p* > 0.05) and in multiple linear regression (*p* = 0.542)	HL did not have a significant association with diabetes medication adherence in bivariate correlation (*p* > 0.05) and in multiple linear regression (*p* = 0.586)
Huang et al., 202,0^70^	NA	• Inadequate health literacy and high medication adherence: 11.2 %; • Inadequate health literacy and low medication adherence: 16.1 %; • Adequate health literacy and high medication adherence: 32.2 %; • Adequate health literacy and low medication adherence: 40.5 %.
Hur et al., 202,0^71^	NA	Health literacy was significantly associated with lower adherence in medication taking subscale (*p* = 0.03)
Hussein et al., 202,1^41^	For each one point of STOFHLA score increase, there is a reduction of 0.3 % in the prevalence of uncontrolled diabetes. Furthermore, when multiple linear regression modeling was conducted, s-TOFHLA was still significantly associated with HbA1c (*p* < 0.002).	NA
Ji M et al., 202,0^40^	Health literacy did not demonstrate significant relationship with glycemic control/HbA1c (*p* = 0.157).	• Participants who had a greater level of health literacy was less likely to have metabolic syndrome (OR = 0.77, 95 % CI [0.60 to 0.97], *p* = 0.028). • Health literacy did not demonstrate significant relationship with self-management behaviors.
Junkhaw et al., 201,9^39^	Critical health literacy was associated with HbA1c levels (*p* < 0.01), others domains were not associated.	NA
Kim et al., 202,0^38^	The direct effect of HL was not significant for HbA1c chance (*p* = 0.019). However, the indirect effects of HL, through self-efficacy, on HbA1c change were statistically significant (*p* = 0.019).	• The direct effect of HL was not significant for quality of life (QoL) (*p* < 0.083). • However, the indirect effects of HL, through self-efficacy, on QoL were statistically significant (*b* = 0.028, SE = 0.006, *p* < 0.001).
Kim et al., 201,9^37^	Analysis of the overall treatment effects of the treatment conditions showed no significant difference among the health literacy treatments groups for HbA1c at 9- and 12-weeks follow-up.	Patients with low health literacy had the highest patient activation level at the 9-week follow-up when provided with the Social Media health literacy intervention group (Adjusted mean 18.01; 95 % CI 70.70 to 85.30] compared to the Telephone health literacy intervention group (adjusted mean 73.72; 95 % CI 68.05 to 79.38) and the usual care control group (adjusted mean 70.27; 95 % CI 64.55 to 75.98).
Klinovszky et al., 202,1^36^	There was no significant relationship between HL level and self-assessed three-month A1C level (*p* = 0.058).	• Significant negative, weak relationship between HL level and the years of diabetes duration (*p* = 0.048), with a significance level of 0.05. • Significant positive, weak correlation between HL level and frequency of self-monitoring blood glucose levels per day (*p* < 0.001), with a significance level of 0.01.
Mann et al., 201,9^48^	NA	• The odds of having microvascular complications were 2.15 times higher for patients with inadequate HL compared with adequate HL in univariate analysis (OR = 2.15, 95 % CI 1.09 to 4.25, *p* = 0.026). The association persisted in multivariate regression, after adjusting for age, sex and duration of diabetes (OR = 2.18, 95 % CI 1.07 to 4.46, *p* = 0.032). • The odds of developing neuropathy were 2.3 times higher for patients with inadequate HL comparated with adequate HL in multivariate regression model (OR = 2.3, 95 % CI 1.05 to 5.2, *p* = 0.037). • The odds of developing retinopathy were 2.21 in cases as compared to control subjects, with borderline significance (*p* = 0.054)
Mashi et al., 201,9^35^	There was no significant relationship between HL level and HbA1c in bivariate analyses and in ordinal regression.	There was no significant relationship between HL level and presence of DM complication in bivariate analyses and in ordinal regression.
Mayberry et al., 201,4^73^	NA	• Non-whites and Hispanics: association between being non-white and Hispanic, as well as speakers of Spanish, and inadequate levels of health literacy (*p* < 0.001). • Income: negative correlation (*p* < 0.001). • Family support measured by DFBC-II higher among patients with inadequate levels of health literacy when compared to those with adequate health literacy (mean 2.8 ± 1.2 vs. 2.2 ± 0.9; *p* = 0.004).
Mehzabin et al., 201,9^34^	s-TOFHLA score was strongly associated with HbA1c in fully adjusted model (*p* < 0.001). For each 1 SD increase in s-TOFHLA score, the HbA1c value would be decreased by 0.60 and thus improve glycemic control.	NA
Nurss et al., 199,7^57^	NA	Difficulty in reading medical texts: more frequent among patients with inadequate levels of HL (16.7 % vs. 54.9 %, *p* < 0.05)
Osborn et al., 20,10^9^	Glycemic control: there was no direct correlation between HL and glycemic control (*r* = 0.04; *p* > 0.05).	• Social support: positive relationship (*r* = −0.2, *p* < 0.05). • Knowledge about diabetes: no relation.
Presley et al., 201,9^74^	NA	Fewer participants with marginal or inadequate health literacy were prescribed an antidepressant than participants with adequate health literacy, 9 % vs. 20 % (*p* = 0.048).
Quartuccio et al., 201,8^33^	Women with low HL had a two-fold higher likelihood of having diabetes compared to those with high HL in the fully adjusted model (OR = 2.2; 95 % CI 1.1 to 4.3). Women with medium HL were also more likely to have diabetes than those with high HL (OR = 1.8, 95 % CI 1.1 to 2.9), though the association was attenuated in the fully adjusted model (OR = 1.6; 95 % CI 1.0‒2.6). HL was not related to diabetes for men (OR = 0.7, 95 % CI 0.4 to 1.2). Higher HL categories were associated with relatively lower mean age adjusted A1C levels for women (6.2 %, 5.9 % and 5.6 % across the low, medium, and high health literacy categories, respectively; *p* < 0.001). Furthermore, this significant trend remained across health literacy categories when women were stratified to those with (*p* = 0.04) and without (*p* = 0.02) diabetes. Similar relationship was found for fasting blood glucose among women overall (fasting blood glucose of 110, 108, and 98 mg/dL in the low, medium, and high HL categories, respectively; *p* < 0.01) For men overall, there was no significant relationship between higher HL categories to lower mean age adjusted A1C (*p* = 0.10) or fasting blood glucose (*p* = 0.68).	NA
Saeed et al., 201,8^32^	s-TOFHLA score was independently associated with HbA1c level after adjustment for age, sex, education, employment and socio-economic status (*p* = 0.001). 29 % of patients with adequate health literacy had tight glycaemic controle (HbA1c < 6.5) (OR = 0.017, 95 % CI 0.0 to 0.15, *p* = 0.00050), compared to 0.73 % of patients with inadequate HL. Almost 92 % of patients with inadequate health literacy had poor glycaemic control, while none of the patients with adequate HL had very poor glycemic control (HbA1c > 8.6).	• 47.4 % of patients with inadequate health literacy had retinopathy (OR = 13.1, 95 % CI: 3 to 57.03, *p* = 0.003) compared with only 6.5 % of subjects with adequate HL. • HL did not have a significant association with nephropathy (OR = 3.5, *p* = 0.274), neuropathy (OR = 1.3, *p* = 0.992) and macrovascular complications (OR = 3.5, *p* = 0.523).
Sarkar et al., 200,6^76^	NA	No significant interactions between self-efficacy and health literacy were documented.
Sarkar et al., 201,1^77^	NA	Inadequate levels of health literacy were associated with: More learning problems (C-index = 0.72, 95 % CI 0.67 to 0.78); More difficulty to read (C-index = 0.68, 95 % CI 0.62 to 0.74).
Sayah et al., 201,4^78^	NA	• Tracking issues: correlation between all 16 screening questions for inadequate levels of health literacy with s-TOFHLA, but with Pearson's correlation ranging from weak to moderate: 0.12 to 0.34. • Years of education: weakly correlated with s-TOFHLA (*r* = 0.27) • Self-efficacy: weakly correlated with s-TOFHLA (*r* = 0.11) • Diabetes knowledge: strongly correlated with s-TOFHLA (*r* = 0.51).
Schillinger et al., 20,02^6^	• Glycated hemoglobin: each additional point in the s-TOFHLA, glycated hemoglobin decreased by 0.02 • Inadequate control: more frequent among patients with inadequate levels of literacy (adjusted OR = 2.03, 95 % CI 1.11‒3.73, *p* = 0.02). • Adequate disease control: no statistically significant difference between the two groups (adjusted OR = 0.57, 95 % CI 0.32‒1.00, *p* = 0.05).	• Retinopathy: more frequent among patients with inadequate level of HL (OR = 2.33, 95 % CI 1.19 to 4.57, *p* = 0.01). • Neurovascular complications: more frequent among patients with inadequate level of HL (OR = 2.71, 95 % CI 1.06 to 6.97, *p* = 0.04). • Lower extremity amputations: no statistically significant difference between the two groups (adjusted OR = 2.48, 95 % CI 0.74 to 8.34, *p* = 0.14). • Nephropathy: no statistically significant difference between the two groups (adjusted OR = 1.71, 95 % CI 0.75 to 3.9, *p* = 0.2). • Coronary heart disease: no statistically significant difference between the two groups (OR = 1.73, 95 % CI 0.83 to 3.6, *p* = 0.15).
Schillinger et al., 200,4^79^	NA	Interpersonal relationship (IPC tool): Domain general clarity: worse among patients with poor literacy in health (OR = 4.54, *p* < 0.01). Explanation of the patient's condition: worse among patients with an inadequate level of health literacy (OR = 3.02, *p* = 0.04). Explanation of the care process (OR = 2.25, *p* < 0.001). Empowerment (OR = 2.05, *p* = 0.02) Decision making (OR = 2.3, *p* < 0.001).
Shigaki et al., 201,0^25^	Glycemic: no relation (*p* = 0.35).	• Physical actives patients: no correlation (*p* = 0.77). • Follow-up diet: positive correlation (*p* = 0.02). This result was not demonstrated in the multivariate analysis: (*p* = 0.23).
Shiyanbola et al., 201,8^59^	NA	Health literacy had a significant moderator effect on the relationship between adherence and concerns beliefs (*p* = 0.014) and threatening illness perceptions (*p* = 0.002). When literacy was separated into numeracy and document literacy, only numeracy moderated the illness perceptions ‒ adherence relationship (*p* = 0.038).
Souza et al., 202,0^31^	HL was independently associated with HbA1c values on the multiple linear regression model (*p* = 0.044).	NA
Swavely et al., 201,4^55^	NA	Knowledge about diabetes, measured by SKILLD tool: Suitable: increase in average accuracy from 64.27 % to 88.34 % in patients with adequate health literacy (*p* < 0.001). Inadequate: increase in average accuracy from 59.86 % to 87.25 % in patients with adequate health literacy (*p* < 0.001).
Tan et al., 202,0^30^	• There was no significant difference in the mean HbA1c readings between the three groups of health literacy levels (*p* = 0.839), however all the three groups of HL levels had poor glycemic control (HbA1c > 6.5 %). • Health literacy was not associated with HbA1c using simple logistic regression analyses (OR = 0.975, 95 % CI 0.826‒1.152, *p* = 0.768).	NA
Tang et al., 200,8^26^	• Glycated hemoglobin: negative correlation. • Univariate analysis: r_s_ −0.32 (*p* < 0.001). • • Multivariate analysis: beta = −0.12 (*p* < 0.001).	NA
Taylor et al., 201,3^29^ (abstract)	Glycemic control: no correlation (*p* = 0.57)	NA
Thurston et al., 201,3^84^ (abstract)	NA	• Age (years): negatively correlated (*p* < 0.01). • Adherence to medication: no correlation. • Remember medication: lower levels of health literacy were associated with greater patient difficulty in remembering their medication.
Thurston et al., 201,5^28^	Glycated hemoglobin: no statistically significant association between health literacy indices and glycated hemoglobin values in bivariate analysis: (*p* > 0.05).	• Knowledge about diabetes: positive correlation on DKQ tool (*p* < 0.001). • Medication adherence, measured by MMAS-8: no correlation. • General and specific diet, physical exercise, measurement of blood glucose and care feet: no correlation.
Tseng et al., 201,7^27^	Glycated hemoglobin: The results for the total effect model showed that health literacy was significantly and negatively associated with HbA1c (*p* < 0.05) with covariates included (knowledge of nutrition in diabetes and stages of changes), whereas the direct effect of health literacy on glycemic control was not significant (*p* = 0.1081).	• Knowledge of nutrition in diabetes plays a crucial role in mediating the relationship between health literacy and stages of change, which in turn results in more favorable glycemic control.
Weinger et al., 201,2^81^ (abstract)	NA	• Adherence to medication: no correlation • Patients with high level of health literacy had little difficulty in remembering their medication.
White et al., 201,6^82^	NA	• The negative impact of higher medical mistrust on perceptions of adequate involvement in the decision-making process during encounters was more pronounced for patients with low health literacy than for those with higher health literacy levels (*p* < 0.001 for the interaction).

DFBC-II, Diabetes Family Behavior Checklist II; DKQ, Diabetes Knowledge Questionnaire; HL, Health literacy; IPC, Interpersonal Processes of Care in Diverse Populations Questionnaire; MMAS-8, 8-item Morisky Medication Adherence Scale; NA, Not Assessed; OR, Odds Ratio; s-TOFHLA, Shortened version of TOFHLA; SMBG, Self-Monitoring Blood Glucose; SKILLD, Spoken Knowledge in Low Literacy in Diabetes; 95 % CI, 95 % Confidence Interval.

The results on health literacy reported by these studies are summarized in Table 2.

### Quality of included studies

Table 1 shows the details of the quality assessment of each included study according to the respective tool. The scores of the 44 cross-sectional studies ranged between 4 and 8 (mean = 6.9). All five clinical trials had the domains bias in selection of participants into the study, bias in classification, and bias in selection of the reported judged as high risk of bias by the Cochrane RoB tool. Two experimental prospective before-and-after scored 6 of 9 points. Two systematic reviews scored 11 of 11 points. Only one qualitative research was included and scored 10 out of 10 points. The authors could not assess the study quality of three congress abstracts included in the review, because there was no instrument validated for that.^28^^,^^29^^,^^49^

## Discussion

To conduct this work, the authors identified 57 studies, mostly analytical cross-sectional surveys, with a reasonable reporting of study quality by the Joanna Briggs Institute checklist for analytical cross-sectional (mean of 6.9 out of 8).

The overall prevalence of inadequate Health Literacy (HL) among patients with T2CM varied widely among the included studies, ranging from 4.7 % to 71.7 %. A systematic review, with 29 studies and 13,457 participants that summarized the prevalence of limited HL in persons with T2DM, showed that the global prevalence of limited health literacy was 34.3 % (95 % CI: 25.8 to 42.8) and the prevalence of limited HL in the USA was 28.9 % (95 % CI: 20.4 to 37.3) .^23^ The study suggests that there is a relationship between lower levels of health literacy and worse blood glucose levels and higher rates of disease complications.

Twenty-six studies evaluated the association between health literacy and glycemic target.^6^^,^^9^^,^^24^, ^25^, ^26^, ^27^, ^28^, ^29^, ^30^, ^31^, ^32^, ^33^, ^34^, ^35^, ^36^, ^37^, ^38^, ^39^, ^40^, ^41^, ^42^, ^43^, ^44^, ^45^, ^46^, ^47^ Ten of them found an inverse association between HL and glycated hemoglobin,^6^^,^^26^^,^^27^^,^^31^, ^32^, ^33^, ^34^^,^^39^^,^^41^^,^^43^ suggesting that inadequate blood glucose levels were more frequent among participants with lower levels of health literacy. Schillinger et al. observed that for each additional point in the s-TOFHLA score, glycated hemoglobin decreased 0.02,^6^ and Hussein et al. observed that for each one-point increase in the s-TOFHLA score, there was a reduction of 0.3 % in the prevalence of uncontrolled diabetes.^41^ Both studies suggest a positive correlation between health literacy and glycemic control. However, the other eleven studies that addressed this subject failed to find any association between these variables. This apparent inconsistency could be explained by methodological differences between the studies, including study design, sample size, and data categorization (continuous versus dichotomous variables).

Four studies investigated the association between HL and diabetes complications in people with T2DM.^6^^,^^32^^,^^48^^,^^52^ Microvascular complications like retinopathy and cerebrovascular complications are more frequent in people with inadequate health literacy. Mann et al. observed that the odds of having these complications were 2.15 times higher for people with inadequate HL when compared with adequate HL: 2.3 times higher for developing neuropathy and 2.21 times higher for developing retinopathy. Chen et al. Showed that each point increase in s-TOFHLA score was associated with 4 % lower odds of being in a higher risk category for foot disease. However, this association did not persist after adjusting for age, sex and other covariates.^52^ The literature shows a direct relation between increased glycated hemoglobin levels and diabetic microvascular complications, with a halving of the risk of retinopathy for each point of glycated hemoglobin that is reduced.^53^,^54^

Five studies investigated the association between health literacy and knowledge about diabetes and found a direct association between these two variables.^24^^,^^27^^,^^55^^,^^56^ And one of these studies suggested a direct correlation between HL and glycated hemoglobin (A1C) levels through diabetes knowledge.^27^ This suggests a better management of the disease in people who have greater knowledge about their condition.^57^

One study investigated the association between health literacy and engagement in diabetes treatment. Although the authors did not find an association, participants with inadequate levels of health literacy had greater difficulty in remembering their medications.^28^ Three other studies investigated the association between health literacy and medication adherence and did not find an association,^42^^,^^49^^,^^58^ but another study showed that HL had a significant moderator effect between adherence and concerns, beliefs, and threatening illness perceptions.^59^

As in diabetes, the prevalence of people with inadequate levels of health literacy also varies widely among persons with chronic musculoskeletal diseases and hypertension.^60^ This variation can be attributed to differences between populations and the instruments used to measure health literacy in the various studies. Moreover, these instruments were created and validated over a very long period of time, and the number of questions used in each instrument varies, which has direct implications on the time needed to complete these questionnaires. For instance, while participants take on average 25 minutes to complete the TOFHLA questionnaire, they take only six minutes to fill in the REALM scale. The many differences among the instruments used to assess health literacy make it difficult to compare the levels of health literacy across different studies.^61^

In addition to the systematic reviews by Abdullah et al. (2019)^23^ and Pashaki et al. (2019)^22^ included in this review, there are other systematic literature reviews already published on the topic that have distinct objectives and methodological differences compared to the present review. However, the results of these studies are consistent with those presented in this review, as they did not meet the inclusion criteria for the current analysis.

Caruso et al. (2017) identified 115 records related to Health Literacy (HL) and highlighted key areas of consensus, such as the definition of HL, measurement tools, and the relationship between diabetes knowledge and HL among patients with T2DM. They also pointed out significant knowledge gaps regarding the relationships between HL and health outcomes, self-efficacy, and the effectiveness of interventions.^62^ Similarly, Marciano et al. (2019) developed a meta-analysis comprising 61 studies focused on the impact of health literacy on diabetes-related outcomes. They found a strong association between health literacy and diabetes knowledge, self-care behaviors, and glycemic control, but noted variability depending on the assessment method used. Their analysis emphasized the need for clarity in using health literacy measures within clinical settings.^63^ Additionally, another systematic literature review by Al Sayah et al. (2013) explored the connections between various dimensions of health literacy and health outcomes, concluding that while there is a positive association between health literacy and diabetes knowledge, the evidence on its impact on clinical outcomes remains inconsistent. This review included 34 publications from 24 studies that met the inclusion criteria.^64^

In comparing the present review with those by Caruso et al. (2017), Marciano et al. (2019), and Al Sayah et al. (2013), the authors highlight distinct methodological approaches and findings related to health literacy measures. Whereas Caruso et al. focused on broad themes and identified knowledge gaps, this review specifically examines the prevalence of inadequate health literacy in patients with T2DM and its direct associations with glycemic control and complications. Al Sayah et al. similarly recognized the relationship between low health literacy and reduced knowledge about diabetes, noting its potential obstacle to improving health outcomes. The authors employed a comprehensive search strategy that included a diverse range of study designs without language or publication date restrictions, contrasting with Marciano et al.'s focus on functional health literacy assessed through performance-based and perception-based tools. The present findings underscore the variability in health literacy as influenced by different assessment tools, geographical settings, and cultural contexts, providing unique insights. While Marciano et al. concentrated on the role of functional health literacy in diabetes management, this review emphasizes the challenges posed by using a variety of health literacy measures and their implications for understanding diabetes-related care processes and outcomes. Additionally, by examining intervention contexts and their settings, the authors offer a nuanced understanding that actionably informs clinical practice, suggesting that improvements in self-care and glycemic control hinge on intervention types and healthcare environments. The detailed examination of specific geographic populations, such as Brazilian cohorts, further distinguishes the present study by highlighting demographic challenges and intervention efficacy. These key differences and comparisons between the present review and previous systematic reviews are summarized in Table 3, which highlights distinct objectives, methodologies, and findings.

**Table 3:** Summary of key systematic reviews and meta-analyses on health literacy in Type 2 Diabetes Mellitus.

Study	Aim	Findings / Conclusions	Methodological Notes
Abdullah et al.; 2019	To summarize and report evidence on the prevalence of limited HL in people with T2DM globally	Reported global prevalence of limited HL at 34.3 %; identified factors associated with heterogeneity in prevalence.	Systematic review of 29 studies, including 13,457 participants. Focused on global estimates; did not explore intervention impacts or geographic comparison.
Al Sayah et al.; 2013	To explore connections between health literacy, numeracy, and health results in individuals diagnosed with diabetes	Consistent evidence showed a positive association between health literacy and diabetes knowledge; limited evidence on clinical outcomes.	Analyzed 723 citations, included 34 publications from 24 studies. Geographically limited to Iran; used region-specific tools; did not assess clinical outcomes.
Butayeva et al.; 2023	Evaluated interventions targeting health literacy in T2DM	Positive influence of health literacy interventions on blood sugar control and self-care; hospital settings most effective.	Analyzed 15 randomized controlled trials, spanning 1997‒2021. Focused on RCTs; examined intervention types.
Caruso et al.; 2017	To identify key themes and knowledge gaps in health literacy	Found areas of consensus on health literacy definitions and tools, identified gaps in associations with health outcomes and effectiveness of interventions.	Identified 115 records; various methodologies included. Exploratory focus; did not quantify associations; broad thematic scope.
Marciano et al.; 2019	Meta-analysis on the relationship between health literacy and diabetes outcomes	Clear associations found between health literacy and diabetes knowledge, self-care behaviors, and glycemic control; role of health literacy assessments varied.	Included 61 studies across diverse populations. Compared functional HL measures (performance-based vs. perception-based); high heterogeneity noted.
Pashaki et al.; 2019	Prevalence of health literacy among Iranian patients with T2DM	Reported high rates of inadequate health literacy; emphasized need for educational interventions.	Synthesis of 8 studies; total participants 2230. Emphasized multidimensional HL (e.g., numeracy); clinical outcome links inconclusive.

HL, Health literacy; T2DM, Type 2 Diabetes Mellitus.

Table 3 also details the systematic reviews included in this analysis (Abdullah et al., 2019; Pashaki et al., 2019; Butayeva et al., 2023), and contrasts them with additional notable reviews that did not meet inclusion criteria but provide relevant context (Caruso et al., 2017; Marciano et al., 2019; Al Sayah et al., 2013). The table emphasizes methodological differences such as study design, geographic coverage, types of tools used to assess health literacy, and clinical focus, reinforcing the novelty and specificity of the present work.

The systematic review by Butayeva et al. (2023) aimed to evaluate how interventions targeting health literacy impact blood sugar control and self-care behaviors in individuals with T2DM. They included fifteen randomized controlled trials focusing on health literacy from 1997 to 2021, all in English. Their findings showed that interventions addressing health literacy positively influenced blood sugar control and self-care practices. Individual and telephone-based interventions showed improvements in blood sugar control and self-care habits, respectively. Community worker-led interventions enhanced diabetes knowledge and self-care, while nurse-led interventions were more successful in controlling blood sugar levels. Hospital settings were more effective for blood sugar control than outpatient settings, with significant HbA1c reductions observed after just three months in hospitals. Notably, interventions focusing on health literacy were particularly beneficial for individuals with diabetes for over 7 years, leading to significant enhancements in self-care practices.^65^ However, implementing health literacy interventions can face several challenges, particularly in resource-limited settings. Common barriers include a lack of trained personnel to effectively deliver health literacy programs, insufficient funding to sustain interventions, and limited access to educational resources for patients. Cultural attitudes toward health and wellness can also impact the acceptance and effectiveness of these interventions. Addressing these challenges is crucial to successfully implementing health literacy interventions and to ensuring their positive impact on diabetes management. For example, in several studies included in this review, the absence of standardized training for community health workers hindered the continuity and scalability of interventions. Limited infrastructure in primary care settings, particularly in low-income regions such as parts of Brazil and Southeast Asia, further constrained program delivery. Cultural barriers ‒ such as misconceptions about diabetes, mistrust of health professionals, and gender dynamics ‒ were also reported as obstacles to patient engagement. To address these issues, some studies adopted culturally tailored materials and peer-education models, which proved effective in improving comprehension and adherence. Solutions such as integrating health literacy training into existing medical and nursing curricula, developing digital tools for patient education, and establishing partnerships with local organizations can enhance the implementation and sustainability of health literacy interventions.^31^^,^^35^^,^^38^^,^^50^^,^^51^^,^^65^

A strong point of this review was the use of an explicit and reproducible methodology to reduce the exclusion of relevant studies and bias. The authors used a broad and sensitive search strategy in all four databases, without language or date of publication limits, the authors did not exclude any type of study design, the authors followed well-defined selection criteria, and two authors independently selected the studies using an online platform (Rayyan) to avoid loss of data and ensure blinding of investigators.^16^ Two investigators working independently also assessed both reporting and methodological study quality, using valid tools. Moreover, the authors reported these findings following the PRISMA statement recommendations.^10^

The authors acknowledge that this review had some limitations, mostly related to the poor methodological and reporting quality of the included studies. Since the authors chose to include only studies involving people with T2DM, these findings are applicable only to this population and cannot be generalized to other types of diabetes. The authors selected only people with T2DM because most patients with Type 1 Diabetes Mellitus (T1DM) would be children or adolescents, and this would make it difficult to evaluate health literacy. The authors also limited these searches to studies that used validated tools for assessing health literacy. The authors deliberately excluded studies that employed less widely known instruments or used subjective measurements of health literacy in order to increase the internal validity of this review and to facilitate comparisons and possible groupings of studies that used the same tool. Most of the included studies were cross-sectional, and only five randomized clinical trials had domain bias in the selection of participants into the study, bias in classification, and bias in the selection of the reported results as high risk of bias by the Cochrane RoB tool, which limits the final quality of the overall evidence. Finally, the studies included in this review presented a large number of outcomes. While this offered pertinent and valid information, it hindered objective comparisons among the included studies.

The present findings indicate that inadequate levels of health literacy are frequent among patients with diabetes and may be related to poor glycemic control and increased risk of some complications. Since disease control and the prevention of diabetic complications depend on self-care, it is very important to evaluate health literacy in people with T2DM. Therefore, a routine assessment of health literacy in this population could lead to the development of strategies to improve this level of literacy and their knowledge about the disease.

These findings indicate that inadequate levels of health literacy are frequent among patients with diabetes and may be related to poor glycemic control and increased risk of some complications. Since disease control and the prevention of diabetic complications depend on self-care, it is very important to evaluate health literacy in people with T2DM. Therefore, a routine assessment of health literacy in this population could lead to the development of strategies to improve this level of literacy and their knowledge about the disease.

Three studies were conducted in Brazil to assess the level of Health Literacy (HL) among patients with diabetes.^31^^,^^50^^,^^51^ The Brazilian studies assessing HL among patients with diabetes reveal significant challenges, particularly among the elderly and those with low social support. Castro et al. (2014) found that a majority, 73.3 %, of participants had adequate HL.^50^ However, Santos et al. (2016) highlighted a stark contrast among elderly patients, with only 15.8 % demonstrating adequate HL, and 73.7 % showing inadequate HL.^51^ Similarly, Souza et al. (2020) found that while 53.6 % of elderly patients with low social support had adequate HL, 46.4 % remained inadequately literate.^31^ These findings from Brazilian studies reflect broader patterns observed in other low- to middle-income countries, where limited health literacy is often linked to structural and social determinants of health. This is further supported by the integration of Human Development Index (HDI) data, which illuminates the socioeconomic forces shaping individuals' capacity to access, understand, and use health information effectively.

The HDI, which reflects key dimensions such as education, income, and life expectancy, serves as a robust indicator of a country's developmental status and its impact on health literacy. The present findings highlight how lower HDI levels may exacerbate challenges in diabetes management by limiting access to education and healthcare resources, ultimately contributing to poorer health outcomes. These insights reinforce the patterns observed in low- to middle-income countries and underscore the broader implications for health literacy interventions in similar settings, emphasizing the importance of contextually adapted strategies that address underlying social and economic inequities.

The authors believe that more comparative clinical studies with good methodology and adequate sample size are needed to assess the effects of improved health literacy on clinical and laboratory outcomes in people with T2DM. The future development and use of a single instrument to assess health literacy, combining the advantages of all existing tools, would facilitate comparisons between different populations. In addition, future studies evaluating different strategies to improve health literacy would be interesting.

## Conclusions

According to the findings of this review, the estimated prevalence of inadequate levels of health literacy in different T2DM populations ranged from 4.7 % to 71.7 % and there appears to be a direct association between the level of health literacy and disease control, as well as an inverse association between health literacy and the risk of developing complications (neurovascular and retinopathy). These findings could justify the routine assessment of health literacy among people with T2DM, as well as the development of strategies to improve their levels of health literacy.

## Ethics approval

The study was approved by the local Ethics Committee (2015/0990).

## Funding

This project was financially supported by São Paulo Research Foundation (FAPESP), grant number 15/13994–0.

## CRediT authorship contribution statement

**Ariel Cesar de Carvalho:** Conceptualization, Investigation, Writing – original draft. **Matheus Tonholo Silva:** Data curation. **Isadora Lagreca Garrafa Treptow:** Data curation. **Carolina de Oliveira Cruz Latorraca:** Methodology, Writing – review & editing. **Rafael Leite Pacheco:** Writing – review & editing. **Victor Alexandre dos Santos Valsecchi:** Writing – review & editing. **Rachel Riera:** Conceptualization, Methodology. **Lucas Leite Cunha:** Supervision, Project administration.

## Declaration of competing interest

The authors declare no conflicts of interest.

## Appendix Supplementary materials

Image, application 1
