# Supplementary material for: Health literacy in patients with Type 2 Diabetes Mellitus: A systematic review
Source: Clinics (Sao Paulo). 2025 Sep 22;80:100774. doi: 10.1016/j.clinsp.2025.100774 (PMC12494563; doi:10.1016/j.clinsp.2025.100774)
Supplement: Supplementary file 1 [file mmc1.docx]

**CLINICS-D-24-00297**

**SUPPLEMENTARY MATERIAL**

**Supplementary Table 1** Search strategy in electronic databases.

| **MEDLINE via Pubmed** | #1 “Diabetes Mellitus, Type 2”[Mesh] OR (Diabetes Mellitus, Type 2) OR (NIDDM) OR (Maturity-Onset Diabetes) OR (Diabetes Mellitus, Noninsulin-Dependent) OR (Diabetes Mellitus, Adult-Onset) OR (Adult-Onset Diabetes Mellitus) OR (Diabetes Mellitus, Adult Onset) OR (Diabetes Mellitus, Ketosis-Resistant) OR (Diabetes Mellitus, Ketosis Resistant) OR (Ketosis-Resistant Diabetes Mellitus) OR (Diabetes Mellitus, Maturity-Onset) OR (Diabetes Mellitus, Maturity Onset) OR (Diabetes Mellitus, Non-Insulin Dependent) OR (Diabetes Mellitus, Non-Insulin-Dependent) OR (Non-Insulin-Dependent Diabetes Mellitus) OR (Diabetes Mellitus, Noninsulin Dependent) OR (Diabetes Mellitus, Slow-Onset) OR (Diabetes Mellitus, Slow Onset) OR (Slow-Onset Diabetes Mellitus) OR (Diabetes Mellitus, Stable) OR (Stable Diabetes Mellitus) OR (Diabetes Mellitus, Type II) OR (Maturity-Onset Diabetes Mellitus) OR (Maturity Onset Diabetes Mellitus) OR (MODY) OR (Type 2 Diabetes Mellitus) OR (Noninsulin-Dependent Diabetes Mellitus) OR “Diabetes Complications”[Mesh] OR (Diabetes Complication) Or (Diabetes-Related Complications) OR (Diabetes Related Complications) OR (Diabetes-Related Complication) OR (Diabetic Complications) OR (Diabetic Complication) OR (Complications of Diabetes Mellitus) OR (Diabetes Mellitus Complication) OR (Diabetes Mellitus Complications)  #2 “Health Literacy”[Mesh] OR (Literacy, Health)  #3 #1 AND #2 |
| --- | --- |
| **LILACS via BVS** | #1: mh:(Diabetes Mellitus Tipo 2) OR mh:(Diabetes Mellitus, Type 2) OR tw:(Diabetes Mellitus de Início no Adulto) OR tw:(Diabetes Mellitus Resistente a Cetose) OR tw:(Diabetes Mellitus não Insulinodependente) OR tw:(Diabetes Mellitus não Insulino-Dependente) OR tw:(Diabetes Mellitus não Dependente de Insulina) OR tw:(Diabetes Mellitus Estável) OR tw:(DMNID) OR tw:(Diabetes Mellitus de Início na Maturidade) OR tw:(MODY) OR tw:(Diabetes Mellitus de Início Gradativo) OR mh:(C18.452.394.750.149*) OR mh:(C19.246.300*) OR mh:(Complicações do Diabetes) OR mh:(Diabetes Complications) OR mh:(Complicaciones de la Diabetes) OR tw:(Complicações da Diabetes) OR tw:(Complicações Diabéticas) OR mh:(C19.246.099*)  #2: mh:(Alfabetização em Saúde) OR mh:(Health Literacy) OR mh:(Alfabetización en Salud) OR tw:(Cultura sobre Saúde) OR tw:(Cultura em Saúde) OR mh:(I02.233.332.186.500*) OR mh:(L01.143.450.500*) OR mh:(N02.421.143.827.407.228.500*) OR mh:(N02.421.726.407.228.500*)  #3: #1 AND #2 |
| **Cochrane Library via Wiley** | #1: MeSH descriptor: [Diabetes Mellitus, Type 2] explode all trees  #2: MeSH descriptor: [Diabetes Complications] explode all trees  #3: MeSH descriptor: [Health Literacy] explode all trees  #4: #1 #2 AND #3 |
| **EMBASE via Elsevier** | #1 ('non-insulin dependent diabetes mellitus'/exp OR ('diabetes mellitus'/exp AND 'health literacy'/exp) OR 'diabetes mellitus type 2' OR 'maturity-onset diabetes' OR 'diabetes mellitus, ketosis-resistant' OR 'ketosis-resistant diabetes mellitus' OR 'diabetes mellitus, non-insulin dependent' OR 'diabetes mellitus, non-insulin-dependent' OR 'non-insulin-dependent diabetes mellitus' OR 'diabetes mellitus, noninsulin dependent' OR 'noninsulin-dependent diabetes mellitus')  #2 ('health literacy'/exp OR 'literacy, health')  #3 #1 AND #2 |

LILACS, Latin-American and Caribbean Literature in Health Science.
